# Prevalence and factors of frailty among community-dwelling older adults: a multimorbidity perspective

**DOI:** 10.3389/fpubh.2026.1854511

**Published:** 2026-08-27

**Authors:** Shubing Feng, Pengpeng Wang, Hongxia Huang, Zhenhong Lin, Yuanhang Liu

**Affiliations:** Guangxi Medical University, Guangxi, China

**Keywords:** activities of daily living, frailty, multimorbidity, multivariate logistic regression, older adults

## Abstract

**Background:**

Frailty is a geriatric syndrome that reduces quality of life and increases social burden. Existing frailty research predominantly focuses on hospitalized or nursing home-residing older adults, while surveys targeting community-dwelling older adults with multimorbidity remain scarce.

**Objective:**

To investigate the prevalence and influencing factors of frailty among community-dwelling older adults with multimorbidity.

**Methods:**

A cross-sectional survey was conducted from May to November 2025. Convenience sampling was adopted to recruit multimorbid older adults from five communities in Nanning, Guangxi. Data were collected using a self-designed general information questionnaire, Mini Nutritional Assessment Short-Form (MNA-SF), Barthel Index (BI), Pittsburgh Sleep Quality Index (PSQI), and Groningen Frailty Indicator (GFI). Univariate analyses and multivariate binary Logistic regression were performed to identify factors associated with frailty.

**Results:**

A total of 346 multimorbid community-dwelling older adults were enrolled, including 183 frail individuals (52.9%) and 163 non-frail individuals (47.1%). Multivariate Logistic regression revealed that age, hospital admission due to acute events in the past year, nutritional status, residential area, primary caregiver type, and activities of daily living (ADL) were independent influencing factors of frailty. Compared with participants aged 60–70 years, the risk of frailty was significantly higher in those aged 70–80 years (OR = 2.644, 95%CI: 1.030–6.790) and ≥80 years (OR = 4.923, 95%CI: 1.870–12.960). Older adults admitted for acute events exhibited higher frailty risk than those without acute admission (OR = 1.841, 95%CI: 1.035–0.274). Rural residents had higher frailty risk than urban counterparts (OR = 0.338, 95%CI: 0.191–0.600). Normal nutritional status (OR = 0.227, 95%CI: 0.155–0.496) and full ADL independence (OR = 0.210, 95%CI: 0.056–0.794) were protective against frailty. Taking self-care as the reference group, frailty risk was markedly lower among older adults cared for by spouses (OR = 0.387, 95%CI: 0.158–0.947), children (OR = 0.354, 95%CI: 0.148–0.845), or professional caregivers (OR = 0.241, 95%CI: 0.072–0.811).

**Conclusion:**

The prevalence of frailty is relatively high among community-dwelling older adults with multimorbidity. Age, nutritional status, residential environment, history of acute-event hospitalization in the past year, primary caregiver type, and ADL capacity are independent risk factors for frailty. Primary community health services should establish integrated screening and intervention systems targeting high-risk groups including advanced-age, malnourished, functionally impaired older adults and those lacking caregiver support.

## 1 Introduction

Accelerated population aging has become a critical public health challenge worldwide. Over the past six decades, the proportion of adults aged ≥60 years globally has risen from 8% to 10%, and this figure is projected to reach 22% within the next four decades, with the number of older adults expanding from 800 million to approximately 2 billion (1).

Frailty is a prevalent geriatric syndrome characterized by diminished physiological reserve and multi-system functional decline, which reduces individuals' resilience to external stressors and elevates risks of falls, disability, hospitalization and all-cause mortality (2). Multimorbidity refers to the coexistence of two or more chronic diseases (3), and its prevalence increases sharply with advancing age. Roughly half of Chinese older adults live with multimorbidity, and this subgroup faces substantially higher risks of functional decline and disability (4).

Frailty and multimorbidity interact bidirectionally. Long-term cumulative damage from multiple chronic conditions depletes physiological reserves and accelerates frailty onset; conversely, frailty further exacerbates disease burden, forming a vicious cycle that impairs older adults' quality of life and health outcomes. Notably, frailty is dynamic and potentially reversible, and early identification and targeted interventions can delay or even reverse its progression (2).

Most existing frailty research focuses on hospitalized patients (5) and nursing home residents (6), Meta-analytic data demonstrated that the pooled frailty prevalence among general community seniors only ranged from 10% to 27%, yet frailty rates could reach over 50% among multimorbid older adults, indicating a disproportionately high frailty burden in this specific subgroup (7), while population-based investigations into community-dwelling multimorbid older adults remain limited. Community-dwelling older adults constitute the majority of the aging population, and their health status directly shapes grassroots public health delivery and the realization of healthy aging goals. The Groningen Frailty Indicator (GFI) is a self-reported multidimensional screening tool that evaluates frailty across physical, cognitive, psychological and social domains. It features concise items, convenient administration and comprehensive assessment, rendering it suitable for community-based surveys (8).

Against this backdrop, the present study recruited community-dwelling multimorbid older adults as participants, adopted the GFI to assess frailty prevalence, and explored its associated influencing factors. Findings aim to provide evidence for early identification of high-risk populations and the formulation of community-based intervention strategies.

## 2 Materials and methods

### 2.1 Study design

This cross-sectional study was conducted between May and November 2025 in five communities of Nanning, Guangxi. Convenience sampling was used to recruit eligible multimorbid older adults.

Inclusion criteria: ① Aged ≥ 60 years; ② Diagnosed with two or more chronic diseases simultaneously; ③ Clear consciousness, capable of completing questionnaires with assistance from investigators or family members; ④ Voluntary participation with signed informed consent.

Exclusion criteria: ① Severe mental disorders, cognitive impairment or verbal communication barriers; ② Permanent bedridden status or complete functional dependence; ③ Unconsciousness.

### 2.2 Data collection

A self-designed general information questionnaire was used to collect demographic and physical health data, including gender, age, marital status, educational attainment, residential environment, number of chronic diseases, primary caregiver type, frailty status, nutritional status, ADL performance and sleep quality.

Face-to-face interviews were conducted by uniformly trained investigators to guarantee data accuracy and integrity. A total of 350 questionnaires were distributed; incomplete questionnaires were excluded, yielding 346 valid responses with an effective response rate of 98.9%.

### 2.3 Research instruments

#### 2.3.1 Self-designed general information questionnaire

Demographic data covered age, gender, marital status, residential environment, primary caregiver, education level, source of income, history of hospital admission due to acute events (acute disease exacerbation, fall-related trauma, psychological trauma, etc.) in the past year, smoking, alcohol consumption, physical activity, chronic disease profiles and medication use. Physical indicators included disease categories, number of chronic illnesses, types of medications, disease duration, height, weight, calf circumference and upper arm circumference.

Smoking status was defined per World Health Organization (WHO) standards: individuals with cumulative or continuous smoking for ≥6 months were classified as smokers (9). Physical activity referred to structured exercise such as jogging and tai chi, excluding routine housework or farm labor. Participants were categorized as frequent exercisers (≥3 times weekly, ≥30 min per session), occasional exercisers (1–2 times weekly, ≥30 min per session), or non-exercisers (< 1 time weekly). Consistent with prior research, polypharmacy was defined as daily use of ≥5 medications (3). Calf circumference was measured at the thickest point of the gastrocnemius muscle; upper arm circumference was measured 10 cm above the elbow crease.

#### 2.3.2 Groningen frailty indicator (GFI)

The GFI was applied to evaluate frailty status (10). The scale contains 15 items covering physical, cognitive, psychological and social domains, each scored 0 or 1, with total scores ranging from 0 to 15; higher scores indicate more severe frailty. A total score ≥4 was defined as frail per standard cut-off criteria.

#### 2.3.3 Mini nutritional assessment short-form (MNA-SF)

Developed by Kaiser et al. (11) in 2019, the MNA-SF serves as an independent nutritional screening tool. It includes six items: weight loss over the past 3 months, body mass index, acute disease/stress in the past 3 months, mobility, neuropsychological problems, and appetite loss/digestive disturbance in the past 3 months. Total scores range from 0 to 14; scores of 0–11 indicate malnutrition or malnutrition risk, and scores of 12–14 represent normal nutritional status.

#### 2.3.4 Barthel index (BI)

The BI is a globally recognized standardized tool for ADL assessment, scoring 10 core daily function items with a total score of 100; higher scores reflect better functional independence (12). Consistent with previous research, BI scores were recoded into three categories for statistical analysis (12): fully independent, requiring partial assistance, and fully dependent. Multi-level dependency strata were merged to stabilize statistical power due to small subgroup sample sizes. The Cronbach's α coefficient of the BI in this study was 0.936.

#### 2.3.5 Pittsburgh sleep quality index (PSQI)

Developed by Buysse et al. (13) in 1989, the PSQI evaluates sleep quality over the preceding month. It comprises 19 items across seven domains: subjective sleep quality, sleep latency, sleep duration, sleep efficiency, sleep disturbances, hypnotic medication use, and daytime dysfunction. The total score ranges from 0 to 21; scores ≤ 7 indicate good sleep quality, and scores ≥8 indicate poor sleep quality. The Cronbach's α coefficient of the PSQI in this study was 0.852.

### 2.4 Statistical analysis

SPSS 27.0 software was used for all statistical analyses. Participants were stratified into two groups based on GFI scores: frail group (GFI ≥ 4) and non-frail group (GFI < 4). Normality tests were performed for all continuous variables. Normally distributed continuous data were presented as mean ± standard deviation and compared via independent samples t-test. All continuous variables in this study exhibited non-normal distribution, thus described as median (25th percentile, 75th percentile) [M(P25, P75)] and compared using the Mann–Whitney U test. Categorical variables were reported as frequencies and percentages, with intergroup comparisons conducted via Chi-square test or Fisher's exact test when appropriate.

Frailty status (0 = non-frail, 1 = frail) was set as the binary dependent variable. Variables with *p* < 0.10 in univariate analyses, clinically meaningful factors identified in prior literature (age, gender, nutritional status, number of chronic diseases), and potential confounders (number of chronic illnesses, polypharmacy) were entered into multivariate binary Logistic regression models to identify independent frailty predictors. Odds ratios (OR) and corresponding 95% confidence intervals (CI) were calculated. Multicollinearity diagnostics were performed, with variance inflation factor (VIF) < 5 indicating no significant collinearity.

Interaction terms were constructed to explore effect modification. To stabilize model performance, age was dichotomized into < 70 years (younger older adults) and ≥70 years (advanced older adults) for interaction analyses. An age × ADL interaction term was incorporated into the regression model to test whether age modified the association between ADL capacity and frailty. Stratified regression analyses were conducted separately for each age subgroup if the interaction term reached statistical significance (*p* < 0.05). The assignment of each variable is detailed in Table 1.

**Table 1:** Variable assignment for regression analysis.

Variable name	Coding and definition	Reference category
Educational attainment	1 = junior high school; 2 = college/bachelor's degree; 3 = high school/technical secondary school; 4 = illiterate; 5 = primary school	Primary school
Primary caregiver	1 = spouse; 2 = children; 3 = professional caregiver; 4 = self-care	Self-care
ADL capacity (BI score)	1 = fully independent; 2 = partial assistance required; 3 = fully dependent	Fully dependent
Age group	0 = ≥ 80 years; 1 = 70–80 years; 2 = 60–70 years	60–70 years
Alcohol consumption	1 = non-drinker; 2 = former drinker; 3 = current drinker	Current drinker
Acute event hospitalization	1 = yes; 0 = no	No
Nutritional status	1 = normal nutrition; 0 = malnutrition	Malnutrition
Residential environment	1 = urban; 0 = rural	Rural
Marital living status	1 = solitary living; 0 = cohabitation	Cohabitation
Polypharmacy	1 = no polypharmacy; 0 = polypharmacy	Polypharmacy
Family history of dementia	1 = absent; 0 = present	Present
Gender	1 = male; 0 = female	Female

All statistical tests were two-tailed, and *p* < 0.05 was defined as statistically significant.

## 3 Results

### 3.1 Basic participant characteristics

Among the 346 participants, 183 (52.9%) were classified as frail and 163 (47.1%) as non-frail. The sample included 153 males (44.2%) and 193 females (55.8%); 139 urban residents (40.2%) and 207 rural residents (59.8%). The median age was 71 (66, 76) years. Stratified by age: 171 participants aged 60–70 years (49.4%), 138 aged 70–80 years (39.9%), and 37 aged ≥80 years (10.7%). A total of 111 participants (32.1%) had polypharmacy, and the median disease duration was 7 (4–12) years.

### 3.2 Univariate analyses of factors associated with frailty

Univariate analyses demonstrated statistically significant intergroup differences (*p* < 0.05) between frail and non-frail participants across the following variables: demographic and lifestyle factors (gender, residential environment, alcohol consumption, education, solitary living), clinical disease factors (history of acute-event hospitalization, family dementia history, polypharmacy), physical function and nutritional indicators (ADL performance, calf circumference, upper arm circumference, primary caregiver type, MNA-SF nutritional status) See Table 2 for details.

**Table 2:** Univariate analyses of factors.

Item	Category	Frail (*n* = 183)	Non-frail (*n* = 163)	χ^2^/*Z* statistic	*p* value
Age	60–70 years	97 (53.0)	74 (45.4)	χ^2^ = 2.000	0.368
	70–80 years	68 (37.2)	70 (42.9)		
	≥80 years	18 (9.8)	19 (11.7)		
Gender	Male	68 (37.2)	85 (52.1)	χ^2^ = 7.852	0.005
	Female	115 (62.8)	78 (47.9)		
Living arrangement	Cohabitation	138 (75.4)	140 (85.9)	χ^2^ = 5.996	0.014
	Solitary living	45 (24.6)	23 (14.1)		
Residential area	Rural	128 (69.9)	79 (48.5)	χ^2^ = 49.42	< 0.001
	Urban	55 (30.1)	84 (51.5)		
Smoking status	Current smoker	23 (12.6)	29 (17.8)	χ^2^ = 1.133	0.568
	Non-smoker	139 (76.0)	111 (66.3)		
	Former smoker	21 (11.5)	23 (14.1)		
Alcohol consumption	Current drinker	22 (12.0)	41 (25.2)	χ^2^ = 10.277	0.006
	Non-drinker	139 (76.0)	108 (66.3)		
	Former drinker	22 (12.0)	14 (8.6)		
Educational attainment	Illiterate	49 (26.8)	29 (17.8)	Z = −3.691	< 0.001
	Primary school	79 (43.2)	57 (35.0)		
	Junior high school	46 (25.1)	48 (29.4)		
	High school/technical secondary school	8 (4.4)	26 (16.0)		
	College/bachelor's degree	1 (0.5)	3 (1.8)		
Acute-event hospitalization in past year	Yes	84 (45.9)	45 (27.6)	χ^2^ = 12.339	< 0.001
	No	99 (54.1)	118 (72.4)		
Family history of dementia	Present	27 (14.8)	11 (6.7)	χ^2^ = 5.652	0.017
	Absent	156 (85.2)	152 (93.3)		
Disease duration (years)	Median (P25,P75)	7 (4, 12)	8 (5, 13)	Z = −1.679	0.093
Polypharmacy (≥5 medications daily)	Yes	73 (39.9)	38 (23.3)	χ^2^ = 10.874	0.001
	No	110 (60.1)	125 (76.7)		
Disease awareness	Very familiar	18 (9.8)	9 (5.5)	*Z* = −0.381	0.703
	Moderately familiar	57 (31.1)	64 (39.3)		
	Neutral	72 (39.3)	61 (37.4)		
	Slightly unfamiliar	20 (10.9)	18 (11.0)		
	Very unfamiliar	16 (8.7)	11 (6.7)		
ADL capacity	Fully independent	108 (59.0)	134 (82.2)	χ^2^ = 23.923	< 0.001
	Partial assistance needed	54 (29.5)	25 (15.3)		
	Fully dependent	21 (11.5)	4 (2.5)		
Calf circumference (cm)	Median (P25,P75)	32.3(30.0, 34.6)	34.0(31.0, 36.0)	*Z* = −3.236	0.001
Upper arm circumference (cm)	Median (P25,P75)	26.0(25.0, 27.5)	27.0(25.0, 28.5)	*Z* = −2.168	0.030
Primary caregiver	Spouse	50 (27.3)	66 (40.5)	χ^2^ = 13.790	0.003
	Children	78 (42.6)	71 (43.6)		
	Professional caregiver	16 (8.7)	12 (7.4)		
	Self-care	39 (21.3)	14 (8.6)		
Sleep quality	Good	127 (69.4)	108 (66.3)	χ^2^ = 0.390	0.532
	Poor	56 (30.6)	55 (33.7)		
Nutritional status (MNA-SF)	Normal nutrition	66 (36.1)	122 (74.8)	χ^2^ = 52.256	< 0.001
	Malnutrition/risk	117 (63.9)	41 (25.2)		

Categorical variables are reported as frequency (percentage). Non-normal continuous variables are presented as median (25th percentile, 75th percentile).

### 3.3 Multivariate logistic regression for frailty

All variables with *p* < 0.10 in univariate analyses, combined with clinically meaningful factors and established frailty predictors (age, disease duration), were entered into the multivariate regression model. Collinearity diagnostics yielded VIF values ranging from 1.012 to 1.157 and tolerance statistics of 0.864–0.988, indicating no severe multicollinearity.

Multivariate Logistic regression identified age, hospital admission due to acute events, nutritional status, residential environment, ADL capacity and primary caregiver type as independent correlates of frailty. Frailty risk increased progressively with age: participants aged 70–80 years (OR = 2.644, 95%CI: 1.030–6.790) and ≥80 years (OR = 4.923, 95%CI: 1.870–12.960) exhibited significantly higher frailty odds relative to the 60–70 years reference group. Individuals with a history of acute-event hospitalization had elevated frailty risk (OR = 1.841, 95%CI: 1.035–0.274). Rural residence was associated with greater frailty risk compared with urban residence (OR = 0.338, 95%CI: 0.191–0.600). Normal nutritional status (OR = 0.227, 95%CI: 0.155–0.496) and full ADL independence (OR = 0.210, 95%CI: 0.056–0.794) exerted protective effects against frailty relative to malnutrition and full ADL dependence, respectively. Relative to self-care, receiving care from spouses (OR = 0.387, 95%CI: 0.158–0.947), children (OR = 0.354, 95%CI: 0.148–0.845), or professional caregivers (OR = 0.241, 95%CI: 0.072–0.811) all significantly reduced frailty risk See Table 3 for details.

**Table 3:** Multivariate logistic regression of factors.

Category	Variable	β coefficient	OR	95%CI	*p* value
Hospital admission for acute events	Yes vs. No	0.610	1.841	1.035–3.274	0.038
Age	≥80 years vs. 60–70 years	1.594	4.923	1.870–12.960	0.001
	70–80 years vs. 60–70 years	0.972	2.644	1.030–6.790	0.043
Nutritional status	Normal nutrition vs. Malnutrition	−1.282	0.227	0.155–0.496	< 0.001
Residential environment	Urban vs. Rural	−1.084	0.338	0.191–0.600	< 0.001
ADL capacity	Fully independent vs. Fully dependent	−1.559	0.210	0.056–0.794	0.021
	Partial assistance needed vs. Fully dependent	−0.802	0.448	0.114–1.763	0.251
Primary caregiver	Spouse vs. Self-care	−0.949	0.387	0.158–0.947	0.038
	Children vs. Self-care	−1.038	0.354	0.148–0.845	0.019
	Professional caregiver vs. Self-care	−1.421	0.241	0.072–0.811	0.022

### 3.4 Interaction effect of age and ADL, stratified analyses

An interaction term between dichotomized age and ADL capacity was constructed on the fully adjusted multivariate regression model to test effect modification. After adjusting for nutritional status and other confounders, the interaction term of age × partial ADL assistance reached statistical significance (OR = 0.288, 95%CI: 0.097–0.855, *p* = 0.025), indicating age modified the association between partial functional limitation and frailty See Table 4 for details.

**Table 4:** Interaction analysis of dichotomized age × ADL capacity.

Variable	Category	β coefficient	OR	95%CI	*p* value
ADL capacity	Fully independent vs. Fully dependent	−0.787	0.455	0.112–1.846	0.270
	Partial assistance needed vs. Fully dependent	1.506	4.510	0.508–40.059	0.176
Interaction term	Age × Fully independent	−0.267	0.766	0.444–1.319	0.336
	Age × Partial assistance needed	−1.244	0.288	0.097–0.855	0.025

Given the significant interaction effect, stratified multivariate Logistic regression models were built separately for participants < 70 years and ≥70 years to clarify subgroup differences in frailty predictors. In the younger subgroup (< 70 years), ADL impairment and malnutrition were significant frailty risk factors, with functional limitation conferring substantially elevated frailty odds (OR = 4.076, *p* = 0.002). Among participants aged ≥70 years, only malnutrition remained a statistically significant predictor (OR = 4.733, *p* < 0.001). These findings confirm advanced age as a core risk factor for frailty See Table 5 for details.

**Table 5:** Stratified multivariate logistic regression analyses.

Variable	β coefficient	OR	95% CI	*p* value
Subgroup<70 years				
ADL impairment	1.405	4.076	1.677–9.907	0.002
Malnutrition	1.457	4.292	2.126–8.664	< 0.001
Subgroup ≥70 years				
ADL impairment	0.408	1.503	0.882–2.561	0.134
Malnutrition	1.555	4.733	2.395–9.354	< 0.001

Frailty status (0 = non-frail, 1 = frail) was set as the dependent variable; separate fully adjusted multivariate logistic regression models were constructed for each age stratum.

## 4 Discussion

### 4.1 Overall frailty profile

Multivariate regression confirmed that age, acute-event hospitalization history, nutritional status, ADL capacity and primary caregiver type independently predicted frailty. The overall frailty prevalence of 52.9% in this sample far exceeds previously reported frailty rates of 9%−15% among general community-dwelling older adults, and is higher than the 18.8% prevalence reported by Cai et al. (7) for multimorbid community elders. This disparity highlights an exceptionally heavy frailty burden in populations living with concurrent multiple chronic diseases. Three key mechanisms may explain this elevated prevalence: first, physiological reserve declines intrinsically with advancing age, rendering older adults inherently vulnerable to frailty; second, persistent multimorbidity gradually depletes multi-system functional reserves, accelerates physiological decline, and exacerbates frailty risk via chronic physical discomfort and financial strain from long-term medical treatment; third, multimorbid older adults often reduce social participation due to physical pain or illness stigma, diminishing social engagement and exacerbating social frailty. Additionally, rural residents constituted 59.8% of the sample, characterized by limited local medical resources and inadequate routine health management, further aggravating frailty susceptibility. Therefore, multimorbid community-dwelling older adults should be prioritized in population-wide frailty screening and intervention programs.

### 4.2 Advanced age as a critical frailty risk factor

The present study revealed that advanced age acts as a risk factor for frailty, which is consistent with previous investigations. Increasing age represents one of the most critical risk factors for frailty (14). The underlying mechanisms may be explained as follows: physiological reserve gradually declines and multisystem function deteriorates with aging, reducing individuals' tolerance to external stressors. Functional impairments in mobility, hearing and vision among older adults further contribute to cognitive decline. Meanwhile, older adults frequently experience chronic low-grade inflammation, which further impairs homeostatic regulation and facilitates the onset and progression of frailty (15). In addition, frailty and multimorbidity are highly overlapped in the older adults population, and the two conditions interact to promote the development of frailty (16). On the other hand, advanced-aged individuals tend to have lower levels of family and social engagement. Deterioration in psychological status, social roles and social functioning may trigger social frailty. The cumulative and synergistic effects of these adverse conditions further accelerate the progression of frailty.

Therefore, priority should be given to older adults of advanced age with multimorbidity in community-based geriatric health management. Early screening and comprehensive interventions are required to delay the incidence and progression of frailty.

### 4.3 Acute-event hospitalization as an independent frailty predictor

The present study indicated that hospital admission resulting from acute events in the previous year constitutes a risk factor for frailty among older adults. Compared with older adults without such hospital admissions, those admitted for acute events exhibited a significantly higher risk of frailty, which is consistent with findings from previous studies (17). Existing evidence suggests a bidirectional relationship between frailty and acute hospitalization. On one hand, older adults with frailty possess reduced physiological reserve, rendering them more vulnerable to acute events requiring hospital admission under stress. On the other hand, acute illness and the subsequent hospitalization process *per se* facilitate the onset and progression of frailty, and the frailty status of older patients tends to deteriorate further during acute hospitalization (18). Moreover, increased disease consumption and marked restriction of daily activities during hospitalization may lead to loss of muscle mass and decline in physical function, thereby accelerating the depletion of physiological reserve (19). In addition, acute illnesses are frequently accompanied by inflammatory responses and metabolic disturbances, which further impair the body's capacity for homeostatic regulation (20). Therefore, hospital admission due to acute events within the past year can, to some extent, reflect poor baseline health status and diminished physiological reserve, serving as an important indicator to identify individuals at risk of frailty.

In view of this, older adults with a recent history of hospital admission for acute events should be prioritized for intervention in clinical and community health management. Early frailty screening and comprehensive assessment should be implemented among this population.

### 4.4 Nutritional status and frailty

Multivariate analyses of the present study revealed that malnutrition is one of the risk factors contributing to frailty, which aligns with previous research findings (21). Mechanistically, malnutrition can drive the initiation and progression of frailty through multiple pathways. First, age-related deterioration of oral health and impaired digestive and absorptive function commonly lead to insufficient energy and protein intake among older adults, resulting in reduced muscle mass and muscle strength, and subsequently sarcopenia. Sarcopenia is recognized as a key driver underlying frailty (22). On the other hand, a bidirectional association may exist between malnutrition and frailty. Malnutrition stemming from inadequate or unbalanced energy intake, poor dietary quality and insufficient nutrient supply acts as a critical risk factor for frailty. Nutritional status also exerts direct and indirect effects on inflammatory responses and oxidative stress, two well-established correlates of frailty. Conversely, individuals with frailty are more susceptible to malnutrition due to limited mobility, poor oral health and gut microbiota dysbiosis. Malnutrition in turn further impairs physical function and accelerates frailty progression (23), creating a self-reinforcing vicious cycle between the two conditions.

Based on these findings, nutritional assessment should be integrated as an essential component of routine frailty screening within geriatric health management. Older adults at nutritional risk ought to be identified at an early stage, and combined interventions consisting of nutritional supplementation and physical activity training should be implemented to delay the onset and progression of frailty.

### 4.5 Association between residential environment and frailty

The present study identified a close association between residential environment and frailty, showing that older adults residing in rural areas had a significantly higher risk of frailty compared with urban counterparts, which is consistent with prior research (24). This disparity can be attributed to social and environmental factors at the community level that modulate the onset and progression of frailty (25), as well as systematic gaps between urban and rural regions in medical resources, accessible health services and health literacy. Rural areas suffer from relatively scarce medical resources and substandard chronic disease management. Meanwhile, rural older adults have limited access to health information and insufficient awareness of disease prevention. In addition, the high proportion of empty-nest older adults in rural communities results in inadequate social support, increasing psychological and social vulnerability and further elevating frailty risk. Although rural older adults engage in abundant daily physical labor, such activity lacks standardized scientific training, accompanied by unbalanced dietary patterns. This state of high physical consumption paired with insufficient nutritional replenishment may accelerate physical functional decline.

Accordingly, grassroots medical service capacity in rural communities should be strengthened. Targeted health education and community-based interventions are recommended to reduce the prevalence of frailty.

### 4.6 ADL capacity and frailty

The results of this study indicated that activities of daily living (ADL) constituted an important influencing factor for frailty, and older adults with full functional independence had a markedly lower frailty risk compared with those fully dependent on others. Prior research has demonstrated that low ADL scores are significantly correlated with frailty in older adults (26), which is consistent with the present findings. The potential underlying mechanism is that ADL reflects individuals' basic physical function; impaired ADL performance generally indicates compromised physical function and depleted physiological reserve. Deterioration in functional capacity reduces daily physical activity, which in turn triggers muscle atrophy, impaired cardiopulmonary function and metabolic disorders, forming a vicious cycle of “functional decline—reduced activity—aggravated frailty” (27, 28).

Accordingly, routine ADL assessment within community geriatric evaluations facilitates early identification of populations at high frailty risk. Targeted functional training and rehabilitation interventions can be implemented to slow the progression of frailty.

### 4.7 Primary caregiver type and frailty

This study found that older adults relying entirely on self-care exhibited a significantly higher risk of frailty compared with those receiving care from spouses, children, domestic workers or professional caregivers, which is consistent with previous research (29). Notably, the “self-care” status in this study does not equate to good health; to a certain extent, it represents a vulnerable group lacking external support who are prone to frailty. Caregiving patterns reflect not only the living conditions of older adults but also insufficient social support and limited access to health resources. On the one hand, inadequate external care support may place older adults at a disadvantage in daily living and chronic disease management, markedly elevating their frailty risk. Although some older adults can independently complete daily routines, they lack emotional comfort, systematic disease management and social engagement. Social support and family bonds exert profound impacts on older adults' health, and insufficient support may increase the likelihood of frailty (30). On the other hand, existing studies have identified spouses as the most immediate source of social support (31). Accordingly, older adults cared for by spouses obtain relatively adequate social support. Nevertheless, spouses are often of similar advanced age with limited physical condition and caregiving capacity, yielding weaker protective effects than care provided by adult children or professional caregivers (32). While some self-caring older adults and those receiving professional care obtain partial assistance, such support remains insufficient in continuity, emotional companionship and individualized health management. Furthermore, long-term caregivers commonly suffer heavy psychological and physical burdens, and such caregiving stress can indirectly impair the health status of care recipients (33). In particular, spouses who act as primary caregivers also face an increased risk of frailty themselves.

Based on the above findings, community geriatric health management should prioritize ostensibly self-sufficient older adults who lack social and emotional support. Early frailty screening and comprehensive assessment should be implemented for this population. Strengthening family support and community-based care service systems can improve the overall support available to older adults and thereby delay the onset and progression of frailty.

### 4.8 Interaction effect of age and ADL capacity

Further interaction analyses of the present study revealed a significant effect modification of age on the association between activities of daily living (ADL) and frailty. The correlation between impaired ADL performance and frailty was statistically significant only among participants younger than 70 years, whereas no significant association was observed in those aged 70 years or older. This finding indicated that the impact of ADL on frailty is age-dependent, which may be attributed to the multi-system functional decline underlying frailty (34) and divergent pathogenetic mechanisms of frailty across age strata. Frailty in advanced age is more likely driven by cumulative deficits across multiple physical domains, including physical function, nutritional status and physiological reserve (35). Functional limitation among relatively younger older adults usually signals depleted physiological reserve and reduced muscle strength, serving as an early marker of frailty with strong predictive value. By contrast, frailty development in the oldest-old is jointly shaped by multimorbidity, chronic inflammation and intrinsic physiological degeneration (36), which weakens the relative predictive effect of ADL. In addition, nutritional status was identified as a stable influencing factor across all age subgroups, highlighting its fundamental role in frailty pathogenesis and emphasizing the essential value of nutritional intervention for frailty prevention and management.

For younger older adults, interventions should prioritize functional training and physical capacity improvement to delay frailty onset. For advanced-aged populations, multi-dimensional strategies integrating nutritional support, chronic disease management and comprehensive care interventions are required to achieve effective frailty control.

## 5 Limitation of the study

This study innovatively targets the understudied population of community-dwelling multimorbid older adults, filling an existing research gap in frailty epidemiology. Several limitations should be acknowledged. First, the cross-sectional design precludes causal inference between identified predictors and frailty; longitudinal cohort studies are required to establish temporal relationships. Second, convenience sampling from a single city limits national generalizability of findings. The primary strength of this research lies in its exclusive focus on multimorbid community elders—a high-risk subgroup disproportionately affected by frailty. Routine integrated frailty screening is recommended for all multimorbid community-dwelling older adults to facilitate early prevention and mitigation of frailty progression.

## 6 Conclusion

This cross-sectional study systematically explored independent frailty predictors among community-dwelling older adults with multimorbidity. Advanced age, history of acute-event hospitalization, rural residence, malnutrition, complete ADL dependence, and absence of external caregivers are independent frailty risk factors, with frailty odds rising progressively with age and peaking in adults ≥80 years. Normal nutritional status, full ADL independence, and care provided by spouses, adult children or professional caregivers significantly reduce frailty risk, with professional care conferring the strongest protective effect. Grassroots community health services should implement stratified screening and intervention strategies targeting high-risk groups including advanced-age, malnourished, functionally impaired and unsupported self-care older adults to reduce population frailty prevalence and promote healthy aging.

## Data Availability

The raw data supporting the conclusions of this article will be made available by the authors, without undue reservation.
